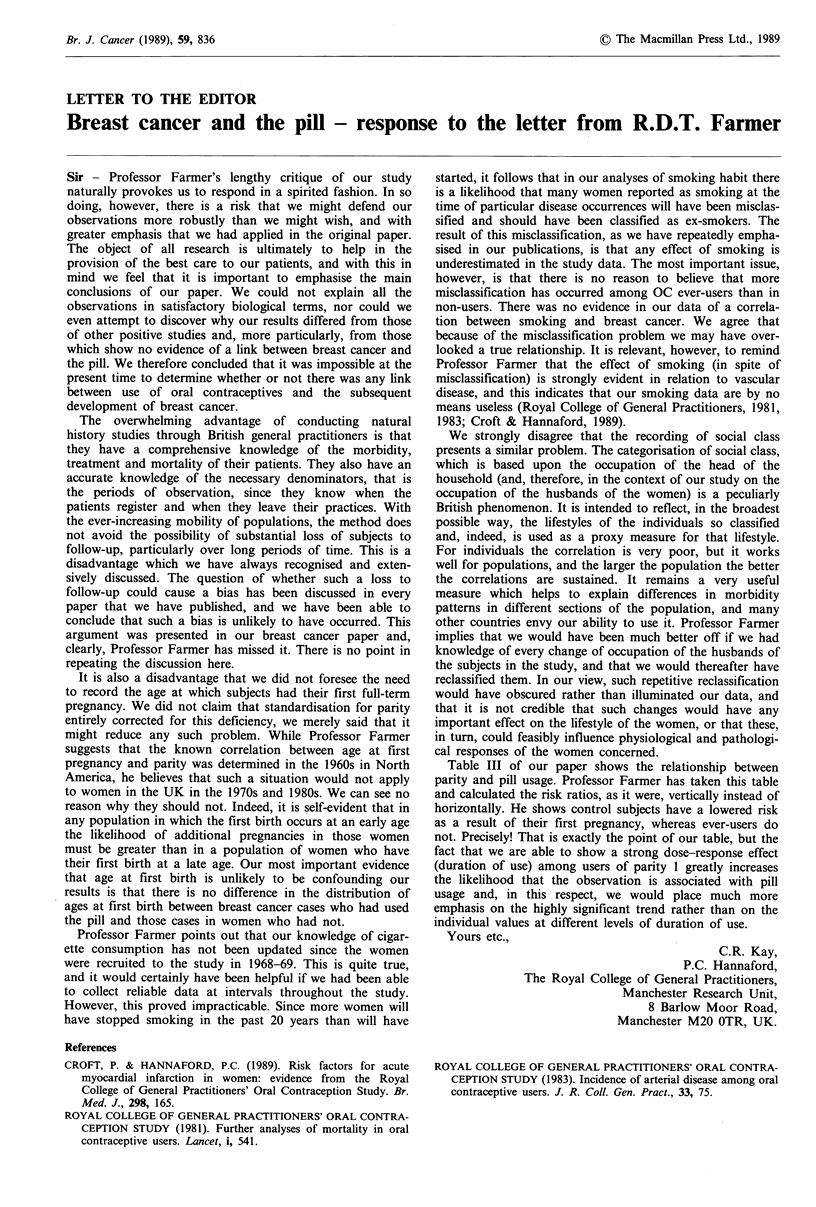# Breast cancer and the pill - response to the letter from R.D.T. Farmer

**Published:** 1989-05

**Authors:** C.R. Kay, P.C. Hannaford


 © The Macmillan Press Ltd., 1989

### LETTER TO THE EDITOR

### Breast cancer and the pill – response to the letter from R.D.T. Farmer

**Sir** – Professor Farmer's lengthy critique of our study naturally provokes us to respond in a spirited fashion. In so doing, however, there is a risk that we might defend our observations more robustly than we might wish, and with greater emphasis that we had applied in the original paper. The object of all research is ultimately to help in the provision of the best care to our patients, and with this in mind we feel that it is important to emphasise the main conclusions of our paper. We could not explain all the observations in satisfactory biological terms, nor could we even attempt to discover why our results differed from those of other positive studies and, more particularly, from those which show no evidence of a link between breast cancer and the pill. We therefore concluded that it was impossible at the present time to determine whether or not there was any link between use of oral contraceptives and the subsequent development of breast cancer.

The overwhelming advantage of conducting natural history studies through British general practitioners is that they have a comprehensive knowledge of the morbidity, treatment and mortality of their patients. They also have an accurate knowledge of the necessary denominators, that is the periods of observation, since they know when the patients register and when they leave their practices. With the ever-increasing mobility of populations, the method does not avoid the possibility of substantial loss of subjects to follow-up, particularly over long periods of time. This is a disadvantage which we have always recognised and extensively discussed. The question of whether such a loss to follow-up could cause a bias has been discussed in every paper that we have published, and we have been able to conclude that such a bias is unlikely to have occurred. This argument was presented in our breast cancer paper and, clearly, Professor Farmer has missed it. There is no point in repeating the discussion here.

It is also a disadvantage that we did not foresee the need to record the age at which subjects had their first full-term pregnancy. We did not claim that standardisation for parity entirely corrected for this deficiency, we merely said that it might reduce any such problem. While Professor Farmer suggests that the known correlation between age at first pregnancy and parity was determined in the 1960s in North America, he believes that such a situation would not apply to women in the UK in the 1970s and 1980s. We can see no reason why they should not. Indeed, it is self-evident that in any population in which the first birth occurs at an early age the likelihood of additional pregnancies in those women must be greater than in a population of women who have their first birth at a late age. Our most important evidence that age at first birth is unlikely to be confounding our results is that there is no difference in the distribution of ages at first birth between breast cancer cases who had used the pill and those cases in women who had not.

Professor Farmer points out that our knowledge of cigarette consumption has not been updated since the women were recruited to the study in 1968–69. This is quite true, and it would certainly have been helpful if we had been able to collect reliable data at intervals throughout the study. However, this proved impracticable. Since more women will have stopped smoking in the past 20 years than will have started, it follows that in our analyses of smoking habit there is a likelihood that many women reported as smoking at the time of particular disease occurrences will have been misclassified and should have been classified as ex-smokers. The result of this misclassification, as we have repeatedly emphasised in our publications, is that any effect of smoking is underestimated in the study data. The most important issue, however, is that there is no reason to believe that more misclassification has occurred among OC ever-users than in non-users. There was no evidence in our data of a correlation between smoking and breast cancer. We agree that because of the misclassification problem we may have overlooked a true relationship. It is relevant, however, to remind Professor Farmer that the effect of smoking (in spite of misclassification) is strongly evident in relation to vascular disease, and this indicates that our smoking data are by no means useless (Royal College of General Practitioners, 1981, 1983; Croft & Hannaford, 1989).

We strongly disagree that the recording of social class presents a similar problem. The categorisation of social class, which is based upon the occupation of the head of the household (and, therefore, in the context of our study on the occupation of the husbands of the women) is a peculiarly British phenomenon. It is intended to reflect, in the broadest possible way, the lifestyles of the individuals so classified and, indeed, is used as a proxy measure for that lifestyle. For individuals the correlation is very poor, but it works well for populations, and the larger the population the better the correlations are sustained. It remains a very useful measure which helps to explain differences in morbidity patterns in different sections of the population, and many other countries envy our ability to use it. Professor Farmer implies that we would have been much better off if we had knowledge of every change of occupation of the husbands of the subjects in the study, and that we would thereafter have reclassified them. In our view, such repetitive reclassification would have obscured rather than illuminated our data, and that it is not credible that such changes would have any important effect on the lifestyle of the women, or that these, in turn, could feasibly influence physiological and pathological responses of the women concerned.

Table III of our paper shows the relationship between parity and pill usage. Professor Farmer has taken this table and calculated the risk ratios, as it were, vertically instead of horizontally. He shows control subjects have a lowered risk as a result of their first pregnancy, whereas ever-users do not. Precisely! That is exactly the point of our table, but the fact that we are able to show a strong dose–response effect (duration of use) among users of parity 1 greatly increases the likelihood that the observation is associated with pill usage and, in this respect, we would place much more emphasis on the highly significant trend rather than on the individual values at different levels of duration of use.

Yours etc.,

C.R. Kay,
P.C. Hannaford,
The Royal College of General Practitioners,
Manchester Research Unit,
8 Barlow Moor Road,
Manchester M20 0TR, UK.

### References

CROFT, P. & HANNAFORD, P.C. (1989). Risk factors for acute myocardial infarction in women: evidence from the Royal College of General Practitioners' Oral Contraception Study. *Br. Med. J.*, **298**, 165.

ROYAL COLLEGE OF GENERAL PRACTITIONERS' ORAL CONTRACEPTION STUDY (1981). Further analyses of mortality in oral contraceptive users. *Lancet*, **i**, 541.

ROYAL COLLEGE OF GENERAL PRACTITIONERS' ORAL CONTRACEPTION STUDY (1983). Incidence of arterial disease among oral contraceptive users. *J. R. Coll. Gen. Pract.*, **33**, 75.